# Insights into the Relationship between the Microstructure and the Catalytic Behavior of Fe_2_(MoO_4_)_3_ during the Ethanolysis of Naomaohu Coal

**DOI:** 10.3390/molecules28186595

**Published:** 2023-09-13

**Authors:** Ting Liu, Xuesong Sun, Yakun Tang, Yue Zhang, Jingmei Liu, Xiaodong Zhou, Xiaohui Li, Lang Liu

**Affiliations:** State Key Laboratory of Chemistry and Utilization of Carbon Based Energy Resources, College of Chemistry, Xinjiang University, Urumqi 830017, China; 17864301835@163.com (X.S.); yktang@xju.edu.cn (Y.T.); yuezhang@xju.edu.cn (Y.Z.); liujm@xju.edu.cn (J.L.); xd_zhou@126.com (X.Z.); lxhui0611@163.com (X.L.)

**Keywords:** Fe_2_(MoO_4_)_3_, catalytic ethanolysis, oxygen-containing compounds, microstructure

## Abstract

Ethanolysis is an effective method to depolymerize weak bonds in lignite under mild conditions, which can result in the production of high-value-added chemicals. However, improving ethanolysis yield and regulating its resulting product distribution is a big challenge. Hence, exploiting highly active catalysts is vital. In this work, Fe_2_(MoO_4_)_3_ catalysts with zero-dimensional nanoparticles, one-dimensional (1D) nanorods, two-dimensional (2D) nanosheets, and three-dimensional (3D) nanoflower structures were successfully prepared and applied in the ethanolysis of Naomaohu coal. The results showed that for all samples, the yield of ethanol-soluble portions (ESP) was significantly improved. The highest yield was obtained for the Fe_2_(MoO_4_)_3_ nanorods, with an increase from 28.84% to 47.68%, and could be attributed to the fact that the Fe_2_(MoO_4_)_3_ nanorods had a higher number of exposed active (100) facets. In addition, the amounts of oxygen-containing compounds, such as ethers, esters, and phenols, increased significantly. The mechanism of ethanolysis catalyzed by the Fe_2_(MoO_4_)_3_ nanorods was also studied using phenylbenzyl ether (BOB) as a model compound. BOB was completely converted at 260 °C after 2 h. It is suggested that Fe_2_(MoO_4_)_3_ nanorods can effectively break the C-O bonds of coal macromolecules, thus promoting the conversion of coal.

## 1. Introduction

Lignite is abundant, accounting for about half of the world’s coal resources, but its application is limited due to its low calorific value, high water content, and poor thermal stability [1,2]. Therefore, exploring ways to achieve the clean and efficient utilization of lignite is of great significance [3]. With respect its structure [4,5,6,7,8], the organic structural units in lignite are connected by weak covalent bonds such as C-O and C-C bonds. Ethanolysis is an efficient coal conversion technology that can break weak covalent bonds in coal at a low temperature. Ethanol can act as a nucleophilic reagent to attack oxygen-containing bridge bonds in coal under supercritical conditions [9,10]. In turn, this promotes the depolymerization of macromolecular structures into small, soluble, organic oxygen-containing molecules, which have high application value and can be used as raw materials for the synthesis of fine chemicals and organic intermediates [11,12,13,14]. Therefore, ethanolysis is an efficient way of converting lignite into chemicals under mild conditions [15,16,17,18,19]. Naomaohu coal (NMHC) is a typical lignite with a high volatile matter and oxygen content. Many studies were carried out on the ethanolysis of Naomaohu Coal. Liang et al. conducted ethanolysis experiments on Naomaohu coal at different temperatures and analyzed the alcoholysis products [20]. Hu et al. conducted ethanolysis experiments on NaoMaoHu coal, analyzed and characterized the alcoholysis products and residues, and deduced the reaction mechanism of ethanolysis [21]. However, the yield of ethanol-soluble portions (ESP) from lignite ethanolysis is limited, and the product distribution is not well regulated. Therefore, it is crucial to explore highly active catalysts for improving the ethanolysis yield and regulating the product distribution.

Strong base catalysts have been widely studied due to their highly catalytic activity in lignite ethanolysis; however, their strong corrosiveness and the cumbersome product handling reduce their practical application prospects [22,23]. In recent years, metal-based catalysts have increasingly gained attention due to their advantageous features of being non-corrosive, low-cost, and easily synthesized [24,25,26,27,28,29]. For example, Li et al. prepared a series of catalysts (MMgAlOx, M = Cu, Zn, Co, and Ni) via the coprecipitation method for the ethanolysis of Xilinguole lignite. The results showed that CuMgAlOx and ZnMgAlOx were very efficient in catalyzing the ethanolysis of XL, allowing for ESP yields of more than 97.0% and the regulation of the distribution of the platform chemicals by altering the catalysts [30]. Ni/HZSM-5 was prepared by Du et al. through a modified deposition precipitation method with a Ni mass ratio ranging from 5% to 20%. The catalytic properties of this catalyst were evaluated by analyzing the catalytic cracking of benzyloxy (BOB) and benzyl ether (BE), and the results showed a high conversion rate and a low amount of rearrangement products. Ni/HZSM-5 was applied in the catalytic ethanolysis of residues from ethanolyzed Xilinguole lignite with a soluble portion yield of 15.1%, indicating that it could successfully cleave C-O bridged bonds without H_2_, producing coal-derived organic chemicals [31].

From the above examples, it can be seen that catalysts play an important role in the depolymerization of coal, and some show an excellent performance, as they can not only increase the yield but also decrease the reaction temperature and change the product distribution. The common key factors thought to affect catalyst activity are particle size and specific surface area. In addition, some studies showed that the microstructure of a catalyst is also important for its catalytic activity. For example, Xie et al. studied the relationship between catalyst microstructure and catalytic behavior during the catalytic conversion of coal. in particular, the effects of different crystal shapes and morphologies on the catalytic activity [32]. Wang et al. reported that lamellar MoS_2_ was more easily inserted into macromolecules, thus improving the catalytic hydrogenation performance [33]. However, the comparison of the effects of microstructures with different dimensions on the a substance has rarely been reported. Thus, the current study investigated the effects of Fe_2_(MoO_4_)_3_ catalysts with different morphologies, achieved by four methods, on the ethanolysis of Naomaohu coal.

In this work, four kinds of Fe_2_(MoO_4_)_3_ catalysts, i.e., with zero-dimensional nanoparticles, one-dimensional (1D) nanorods, two-dimensional (2D) nanosheets, and three-dimensional (3D) nanoflower structures, were prepared by different methods. The morphology and size of the samples were characterized by scanning electron microscope (SEM), and their crystal structure was determined by X-ray diffraction (XRD). The catalysts were applied to the ethanolysis of NMHC, and their mechanism of action was investigated using model compounds. The product distribution of the catalytic ethanolysis was characterized and analyzed using a gas chromatographer–mass spectrometer (GC-MS), and possible reaction pathways of catalytic ethanolysis are proposed. The relationship between morphology and catalytic performance was investigated systematically.

## 2. Results and Discussion

### 2.1. Catalyst Characterizations

**Figure 1:**
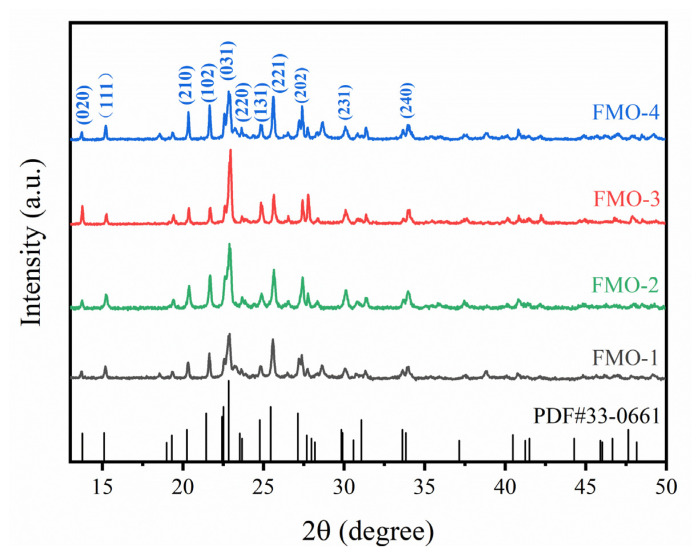
XRD patterns of the samples.

Figure 1 shows the XRD patterns of Fe_2_(MoO_4_)_3_ prepared by different methods. As can be seen, all the observed diffraction peaks of FMO-1, FMO-2, FMO-3, and FMO-4 could be indexed to the monoclinic phase of iron molybdate, with lattice constants a = 9.3300 Å, b = 12.8680 Å, and c = 9.2420 Å, along with α = γ = β = 90°, which matched well with the standard XRD pattern (JCPDS card No. 33-0661), indicating that Fe_2_(MoO_4_)_3_ was successfully prepared. The diffractions peaks at 18.5° observed for FMO-1 and FMO-4 were indexed to FeMoO_4_ (JCPDS card No. 22-0629).

**Figure 2:**
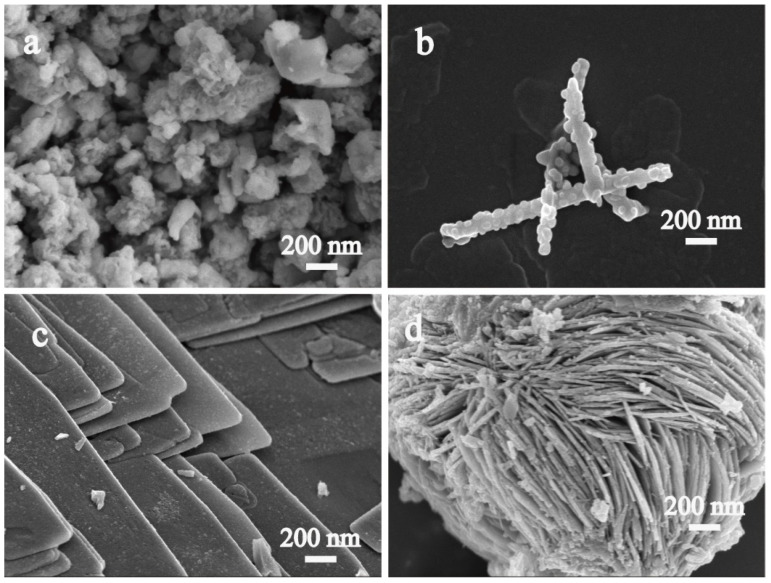
The SEM images of the Fe_2_(MoO_4_)_3_ samples; (**a**) FMO-1, (**b**) FMO-2, (**c**) FMO-3, (**d**) FMO-4.

The morphologies of the prepared Fe_2_(MoO_4_)_3_ samples were investigated by SEM, and the results are shown in Figure 2. It was found that FMO-1 consisted of nanoparticles (Figure 2a). FMO-2 was characterized by nanorods consisting of nanoparticles, whose average diameter was 150 nm (Figure 2b). FMO-3 consisted of nanosheets with a regular shape and a large size, as shown in Figure 2c. In addition, Figure 2d shows the morphology of FMO-4, in which a stratified structure made of stacked nanosheets can be clearly observed. Compared with FMO-3, FMO-4 had more stacked layers and showed a 3D flower shape.

**Figure 3:**
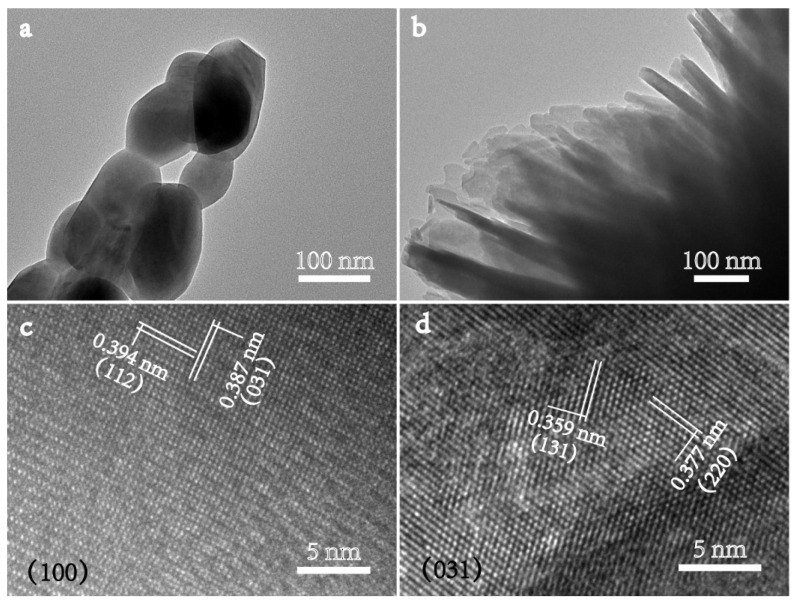
TEM images of (**a**) FMO-2, (**b**) FMO-4; HRTEM images of (**c**) FMO-2, (**d**) FMO-4.

**Figure 4:**
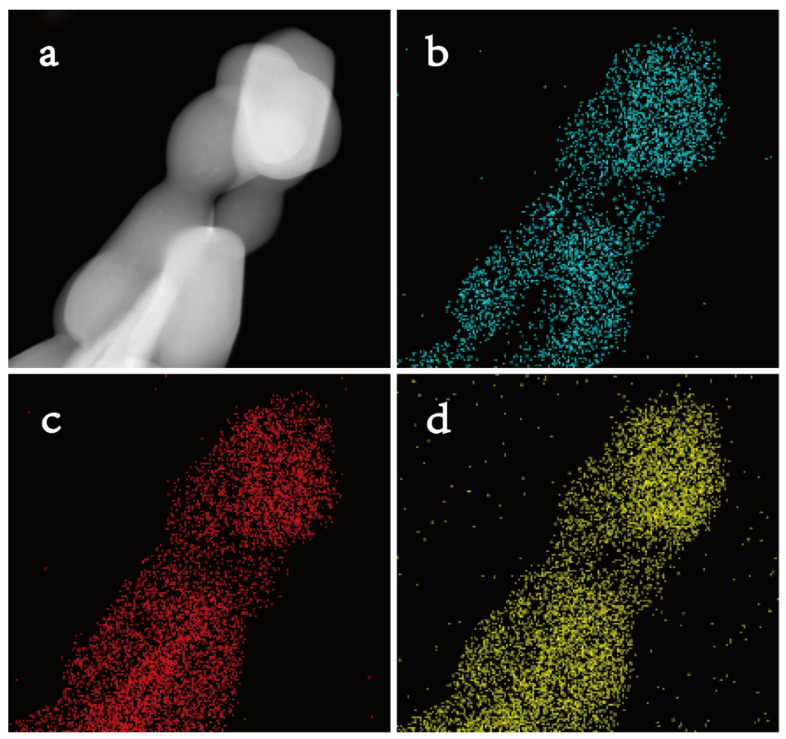
EDS elemental mappings of FMO-2. (**a**) TEM image and (**b**) Fe, (**c**) Mo, and (**d**) O elements.

The crystal structures of the FMO-2 and FMO-4 samples were characterized by TEM. In Figure 3a, it can be seen that FMO-2 consisted of nanoparticles, and the diameter of the nanorods was around 150 nm. Figure 3b shows that FMO-4 consisted of stacked nanosheets. In addition, Figure 3c,d shows the HRTEM images of FMO-2 and FMO-4, respectively, along with the lattice fringes of nanorods and nanoflowers. The lattice spacings indicated in Figure 3c are about 0.387 and 0.394 nm, corresponding to the (031) and (112) crystal planes. The lattice spacings indicated in Figure 3d are about 0.359 and 0.377 nm, corresponding to the (131) and (220) crystal planes of the monoclinic Fe_2_(MoO_4_)_3_ phase. In Figure 3c,d, it can be seen that the (100) plane of FMO-2 is exposed to the maximum extent, and the maximum exposed surface of FMO-4 is the (031) plane. To investigate the elemental distribution, we performed the EDS mapping of individual Fe_2_(MoO_4_)_3_ nanorods. In Figure 4, it can be seen that the Fe, Mo, and O elements were uniformly distributed throughout the nanorod region, thereby demonstrating that FMO-2 was produced by the growth of Fe_2_(MoO_4_)_3_ nanoparticles.

**Figure 5:**
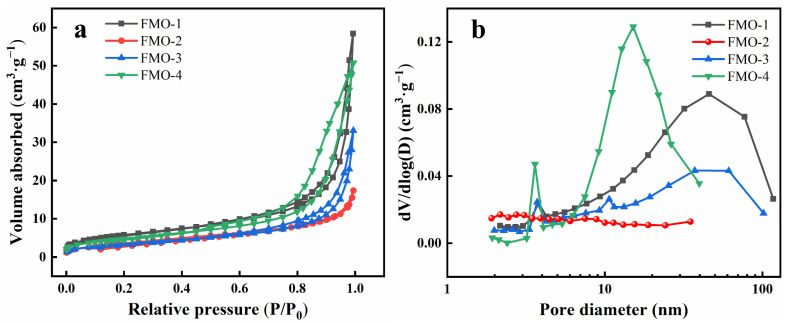
N_2_ adsorption–desorption isotherms (**a**) and pore size distribution (**b**) of FMO-1, FMO-2, FMO-3, and FMO-4.

The four samples were characterized for N_2_ adsorption. The results are shown in Figure 5. In the Figure 5, it can be seen that FMO-1, FMO-3, and FMO-4 presented classical type IV isotherms, indicating that they had mesoporous structures. Among them, FMO-4 presented a larger hysteresis loop, indicating a more mesoporous structure. This was also evidenced by the pore size distribution curve of FMO-4. FMO-2 presented a typical III type isotherm, and its pore structure was dominated by mesopores, with a few micropores. The FMO-2 sample was characterized by NH_3_ temperature-programmed desorption (NH_3_-TPD), and the result are shown in Figure S1. In Figure S1, the desorption peaks appear at 100 °C and 250 °C in the FMO-2 curve and were assigned to NH_3_ desorption from weak and moderately strong acid sites, respectively. The intensity of the desorption peaks of moderately strong acid sites was relatively weak, indicating a weak acidity for FMO-2.

**Figure 6:**
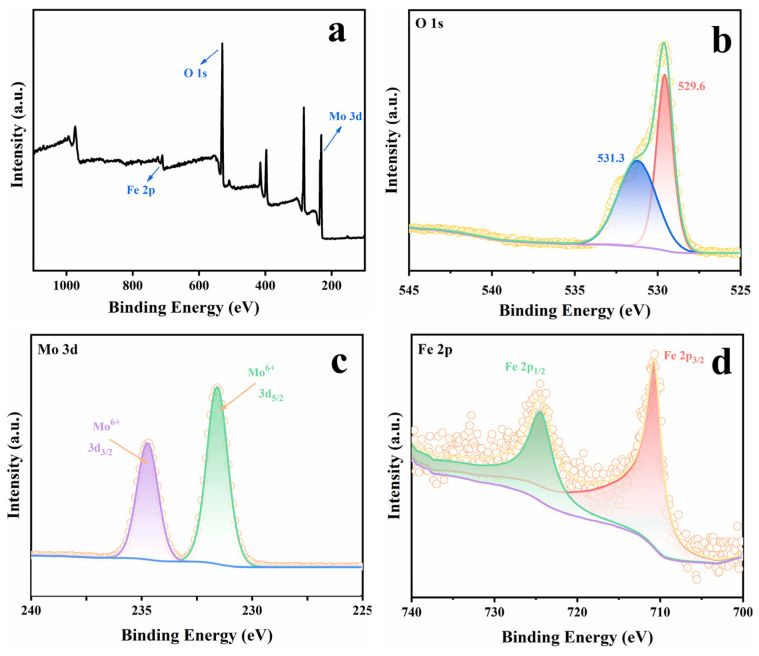
(**a**) XPS spectrum of Fe_2_(MoO_4_)_3_ nanorods, (**b**) O 1s XPS spectrum, (**c**) Mo 3d XPS spectrum, and (**d**) Fe 2p XPS spectrum.

XPS characterization of Fe_2_(MoO_4_)_3_ nanorods was performed to identify its chemical valence and chemical elements. The XPS survey spectra showed that the Fe, Mo, and O elements existed in FMO-2, and the signals of Fe 2p, Mo 3d, and O 1s were clearly observed (Figure 6a). The two peaks of O located near 531.1 and 529.6 eV could be identified from the curve of the O 1s region (Figure 6b). The binding energy peaks of Mo 3d at 234.8 and 231.6 eV belonged to the orbital electrons of 3d_5/2_ and 3d_3/2_ of Mo^6+^, respectively [34,35] (Figure 6c). The curves of Fe 2p were fitted into two peaks of Fe 2p_3/2_ and Fe 2p_1/2_ at 724.6 and 710.8 eV, respectively, revealing the presence of Fe^3+^ [36,37] (Figure 6d).

### 2.2. ESP Yields of Ethanolysis

**Figure 7:**
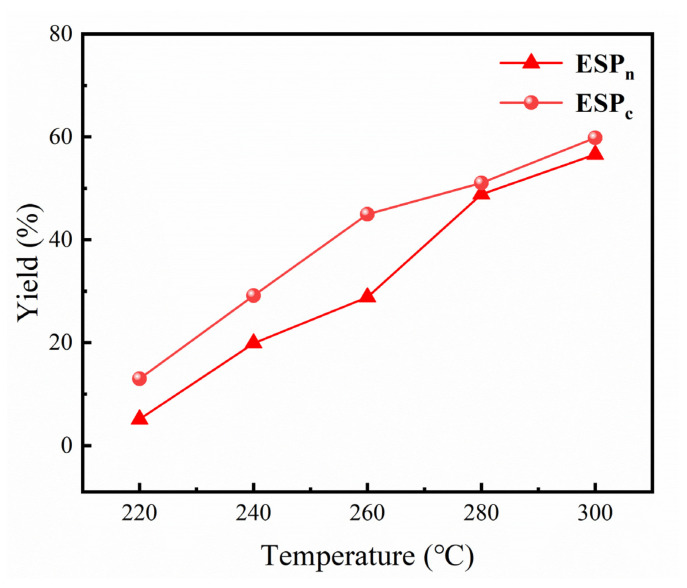
Effect of ethanolysis reaction temperature and catalyst on the ESP yield.

The effect of the Fe_2_(MoO_4_)_3_ catalyst on the ethanolysis of NMHC at different temperature was investigated. As Figure 7 shows, with the addition of FMO-1, the ESP yield increased significantly from 220 °C to 260 °C. However, at temperatures of 280 and 300 °C, the yield of ESP stopped increasing due to a large amount of gas produced by the catalytic cracking effect of FMO-1 above 280 °C. The largest increase in ESP yield, from 28.84% to 44.98%, was at 260 °C after adding the catalyst. Therefore, 260 °C was chosen as the temperature for catalytic ethanolysis.

**Figure 8:**
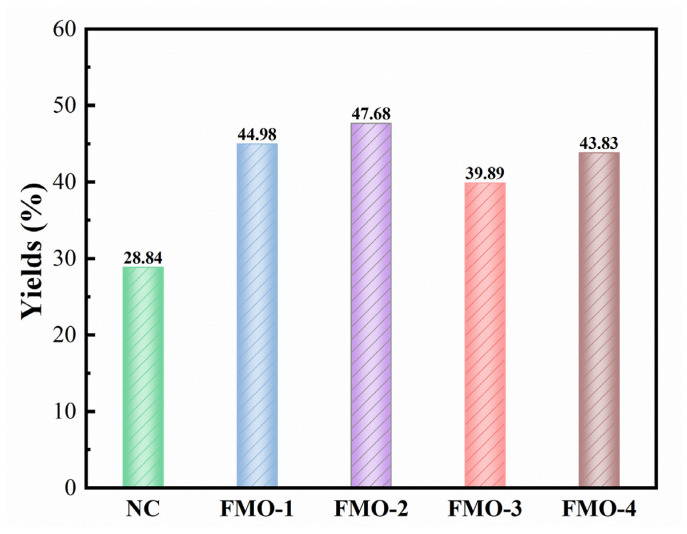
Effect of different catalysts on ESP yield at 260 °C.

Next, the catalytic activities of Fe_2_(MoO_4_)_3_ catalysts with different morphologies during ethanolysis were investigated at 260 °C. The results indicated an obvious improvement in ESP yield after adding the catalysts (Figure 8). Among the four catalysts, FMO-2 showed the best catalytic activity, with the yield of ESP increasing from 28.84% to 47.68%. To investigate the mechanism underlying the different catalyst activity of the Fe_2_(MoO_4_)_3_ catalysts, BET was conducted on the four samples. The results are shown in Table S1. According to Table S1, the specific surface areas of FMO-1, FMO-2, FMO-3, and FMO-4 were 21.0127, 14.0543, 12.9181, and 17.6475 m^2^/g, respectively. It is worth noting that the FMO-2 sample had the best catalytic activity, but its specific surface areas was not satisfying. According to the literature, the active surface of Fe_2_(MoO_4_)_3_ consists of (100) facets [38,39,40]. The HRTEM results showed that the (100) facets of FMO-2 were maximally exposed, and the maximum exposed surface of FMO-4 consisted of (031) facets. This is maybe why even though the specific surface area of FMO-2 was small, its catalytic activity was better than that of FMO-4. Therefore, the exposed surface plays an important role in the catalytic ethanolysis reaction.

### 2.3. Characterizations of ESP and Ethanolysis Residues (ER)

**Figure 9:**
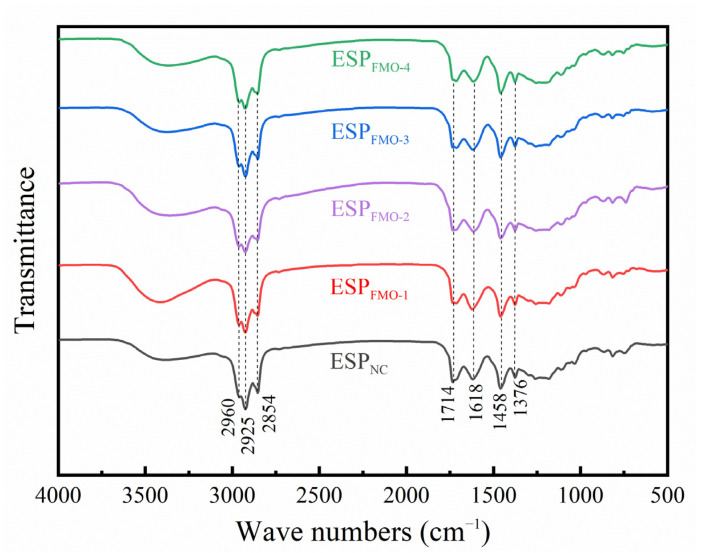
FTIR spectra of ESP.

As shown in Figure 9, the FTIR spectra of ESP with different catalysts were basically the same. The absorbance peak around 3380 cm^−1^ was attributed to the OH bond. Compared with the condition without catalysts, the content of OH bonds in the ESP increased, which indicated that the C-O bonds were broken when the catalysts were added to the ethanolysis reaction. The absorbance peaks around 2854, 2925, 2960, 1458, and 1376 cm^−1^ were assigned to aliphatic moieties, such as alcohols and esters. The absorbance peak around 1714 cm^−1^ was caused by the stretching vibration of C=O in esters, aldehydes, and ketones, while the absorption peak of 1618 cm^−1^ was attributed to the C=C stretching vibration of the aromatic ring. From the FTIR, we could also see that the ESP were rich in oxygen-containing compounds, which was analyzed further by GC-MS.

To investigate the effect of the catalysts on product distribution, the ESP were analyzed by GC-MS. A total of 96 compounds were detected among the ESP of ethanolysis at 260 °C (ESP_NC_), and 138 compounds were detected among the ESP of ethanolysis catalyzed by FMO-2 at 260 °C (ESP_FMO-2_). They were classified as aliphatic hydrocarbons, aromatic hydrocarbons, esters, ethers, alcohols, acids, ketones, phenols, and heteroatomic compounds.

**Figure 10:**
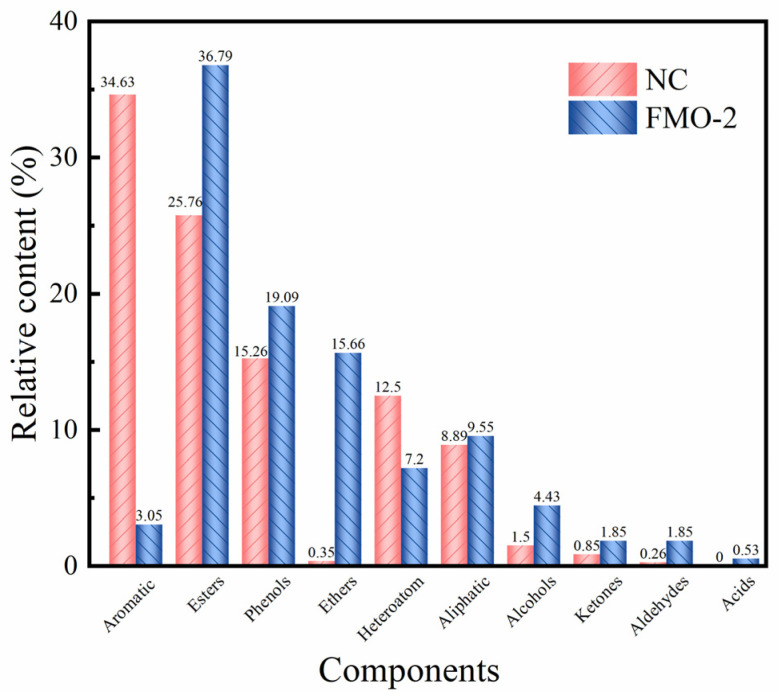
Compound distribution among ESP_NC_ and ESP_FMO-2_.

Figure 10 shows the compound distribution of ESP_NC_ and ESP_FMO-2_. After the addition of FMO-2, the content of aromatic hydrocarbon was significantly reduced, from 34.63% to 3.05%, while the contents of esters, phenols, ethers, ketones, and other organic oxygen-containing compounds, which are considered important platform chemicals, increased significantly. The content of esters, mainly, fatty acid ethyl esters, increased from 25.76% to 35.79% (Table 1). These fatty acid ethyl ester compounds formed in two ways: by the esterification of ethanol with carboxylic acid in NMHC and the transesterification of ethanol with esters originally existing in NMHC. However, according to the FTIR (Figure S2) of NMHC, the content of carboxylic acid was low, which means that the ethyl compounds in the ESPs were mainly generated by the transesterification of ethanol with esters [10,21]. Therefore, FMO-2 as a catalyst can promote transesterification in ethanolysis. According to Table 2, most of the esters among the catalytic ethanolysis products have high molecular weights and are typically used in the preparation of fragrances and flavors.

The phenolic compounds in the ESP are shown in Table 2. The content of phenolic compounds increased from 15.26% to 19.09% after the addition of FMO-2. The phenolic compounds in the ESP were generated in two ways: either by dissolving the original phenolic compounds of NMHC in ethanol or by cleaving the Ar-CH_2_-O-Ar bond in NMHC [41]. The addition of the catalyst resulted in an increase in the content and type of phenolic compounds; so, the second rote should be their main way of production. The phenol compound content was 13.32% after the addition of FMO-2. Phenol is a raw material that has a high economic value and is used in the synthesis of phenolic resins [42]. Therefore, ethanolysis from lignite can be further developed for future phenol production.

**Scheme 1:**
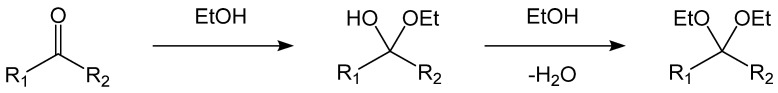
Possible pathways for ether production from catalytic ethanolysis.

The ethers in the ESP increased from 0.35% to 15.66% after adding FMO-2. All identified ethers in the ESP are shown in Table S7. As can be seen, the ether compounds in the ESP mainly consisted of acetals with two ether bonds. Particularly, the content of 1,1-diethoxy-2-methylpropane (special ether), which was the most abundant compound in the ESP, was as high as 9.68%. There are two possible ways of ethers formation. One is the self-reaction of ethanol, and the other is the destruction of the C=O bonds in the coal by ethanol. The second possible formation mechanism is shown in Scheme 1; here, the ethanol, as a nucleophilic reagent, attacks the carbon atom attached to the oxygen to form a hemiacetal. The hemiacetal is unstable and continues to react with ethanol to form acetals. In the classification of the products detected by GC-MS, acetal was considered as a special ether. The FTIR analysis results also supported this conclusion. Above all, FMO-2 could change the product distribution of NMHC ethanolysis.

**Figure 11:**
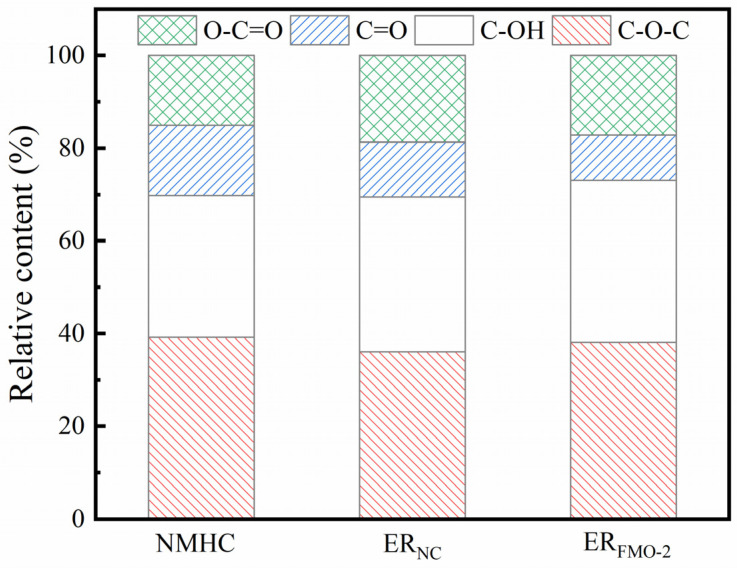
Relative content of oxygen-containing functional groups for NMHC and ER.

The FTIR of NMHC and ER is shown in Figure S2. In the FTIR spectra, the wavenumber range of 1000–1800 cm^−1^ is mainly the absorption vibration region of oxygen-containing functional groups [20]. There are four types of oxygen-containing functional groups in coal: O-C=O, C=O, C-OH, and C-O-C. To investigate the changes of oxygen-containing functional groups after the addition of the catalysts in the process of ethanol hydrolysis, 18 subcurves were fitted in the FTIR spectral region of 1000–1800 cm^−1^ (Figures S3–S5). The variation of oxygen-containing functional groups was calculated according to the fitted peak area, and the results are presented in Tables S10–S12. The total relative content of four oxygen-containing functional groups was set as 100%. The relative content change of these functional groups in NMHC and ER is displayed in Figure 11. As shown in Figure 10, C=O was broken after ethanolysis, and its relative content decreased from 15.1% in NMHC to 9.8% in ER_FMO-2_. The results indicated that the addition of ferric molybdate in the process of ethanolysis promoted the break of C=O bonds in coal.

### 2.4. Catalytic Activity in the Cleaving of BOB

BOB was used as a model compound to explore the reaction mechanism during catalytic ethanolysis. The effects of different temperatures on the catalytic cracking of BOB by FMO-2 were investigated. As shown in Table 3, the conversion of BOB was only 46.77% at 220 °C and reached 92.39% at 240 °C. The conversion of BOB at 260 °C and 280 °C was 100%. Toluene, phenol, (ethoxymrethyl) benzene, 2-ethylphenol, and 2,5-diethylphenol were detected among the products. With the increase in the temperature, the amount of toluene among the products was basically unchanged, while the amount of phenol gradually decreased, and those of 2-ethylphenol and 2,5-diethylphenol gradually increased. The conversion rate was 100% at 260 °C, and there was a lower quantity of rearrangement products. Thus, we chose 260 °C as the temperature at which to study the catalytic ethanolysis of NMHC.

**Scheme 2:**
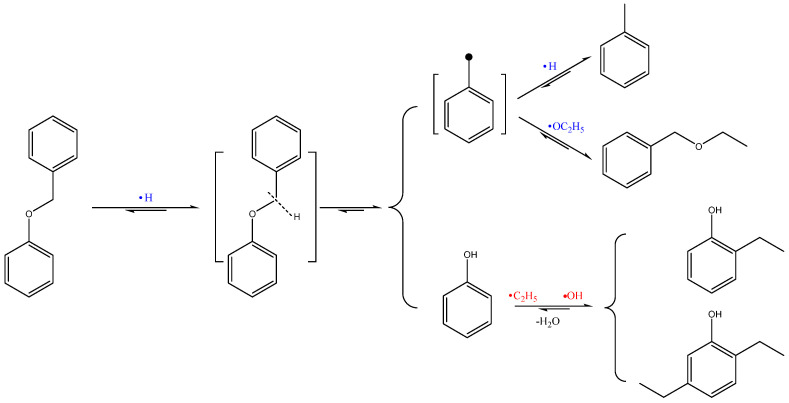
Possible pathways of the catalytic ethanolysis of BOB in the presence of FMO-2.

The possible reaction pathways of FMO-2-catalyzed BOB ethanolysis are shown in Scheme 2. As can be seen, ethanol activated on Fe_2_(MoO_4_)_3_ nanorods could release H· and CH_3_CH_2_O·, and the breaking of the C-O bond in BOB produced phenol and benzyl. Benzyl reacted with H· and CH_3_CH_2_O· to form toluene and (ethoxymrethyl) benzene, respectively. As the temperature increased, ethanol was activated to OH· and CH_3_CH_2_·, and phenol reacted with CH_3_CH_2_· to form 2-ethylphenol and 2,5-diethylphenol. Meanwhile, 2-ethylphenol and 2,5-diethylphenol were detected among the products of FMO-2-catalyzed ethanolysis. Therefore, during catalytic ethanolysis, ethanol may also be activated to form OH· and CH_3_CH_2_·.

### 2.5. Possible Mechanisms of NMHC Catalytic Ethanolysis

**Scheme 3:**
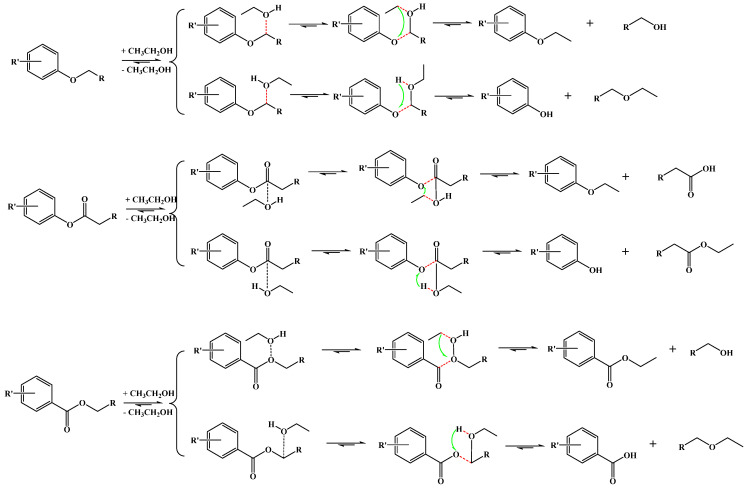
Possible pathways for the catalytic ethanolysis of NMHC in the presence of FMO-2.

A possible reaction pathway for catalytic ethanolysis in the presence of FMO-2 was inferred from the mechanism of the reaction with the model compound. The possible reaction pathway for NMHC catalytic ethanolysis is shown in Scheme 3. R denotes an alkyl or aryl group, and R’ denotes an alkyl group. Based on the experimental results obtained with the model compounds, ethanol activated on FMO-2 could release H· and CH_3_CH_2_O·, ethanol could also be activated to form OH· and CH_3_CH_2_·. In addition, FMO-2 could reduce the activation energy of the C-O bond breakage, thus promoting C-O bond breakage in coal [43]. As we can see in the Scheme 3, ethanol acted as nucleophilic reagents to attack the C-O bonds in NMHC. Oxygen in OH· and CH_3_CH_2_O·, as a nucleophile atom, attacked the C-O bond in NMHC to produce oxygen-containing organic compounds such as alkanols, phenols, esters, and ethers. FMO-2 weakened the C-O and O-H bonds in ethanol, making the oxygen in ethanol more reactive and thus promoting the formation of oxygen-containing compounds.

## 3. Materials and Methods

### 3.1. Materials

Lignite was collected in Naomaohu, Xinjiang, China, and denoted as NMHC. The coal was pulverized and passed through a 200-mesh sieve; its proximate and ultimate analyses are shown in Table 4. The pulverized coal samples were dried in an oven at 60 °C for 48 h. Fe(NO_3_)_3_·9H_2_O, (NH_4_)_6_Mo_7_O_24_·7H_2_O, Na_2_MoO_4_·2H_2_O, H_2_O_2_, HNO_3_, and phenylbenzyl ether (BOB) were used as analytical reagents and were purchased from Sinopharm Chemical Reagent.

### 3.2. Synthesis of the Fe_2_(MoO_4_)_3_ Samples

Fe_2_(MoO_4_)_3_ nanoparticles: ammonium molybdate (1.05 g), ferric nitrate (0.60 g), zinc chloride (5.45 g), and potassium chloride (2.98 g) were ground for 1 h in a ball milling tank. Then, the mixture was calcined at 320 °C with 2 h. A green product was obtained after washing with DI water and drying (denoted as FMO-1).

Fe_2_(MoO_4_)_3_ nanorods: we mixed 7.20 g of molybdenum trioxide powder with 50 mL of 30% hydrogen peroxide by stirring. Next, 27 mL concentrated nitric acid and 170 mL deionized water were added to the above solution and left for 4 days. Afterwards, 60 mL of the mixture was transferred to 100 mL of the lining of PTEE and reacted at 170 °C for 12 h. At the end of the reaction, the precipitate was separated by centrifugation to obtain molybdenum trioxide nanorods. The prepared molybdenum trioxide nanorods were stirred vigorously and dispersed in 100 mL of deionized water, Next, 0.90 g of ferric nitrate was added to the suspension, after which it was stirred at 50 °C for 2 h. After calcination at 500 °C for 4 h, the product was obtained (denoted as FMO-2).

Fe_2_(MoO_4_)_3_ nanosheet: sodium molybdate (1.84 g) and ferric nitrate (2.05 g) were dissolved in 60 mL of DI water. The solution was transferred into the lining of PTEE and heated at 140 °C for 8 h. Then, it was washed and dried to obtain a green product (denoted as FMO-3).

Fe_2_(MoO_4_)_3_ nanoflower: ammonium molybdate (0.37 g) and ferric nitrate (0.56 g) were dissolved in a solvent consisting of 30 mL of DI water and 30 mL of absolute ethanol, within a round-bottom flask. Then, the round-bottom flask was transferred to a microwave reactor and reacted at 90 °C for 30 min. A product was obtained after cleaning and drying the material and was denoted as FMO-4.

### 3.3. Ethanolysis

The ethanolysis experiment was conducted in a 100 cm^3^ high-pressure mechanical agitation batch reactor, in which 3.00 g of dried pulverized coal sample and 60 mL of ethanol, accurately weighed were placed. The reactor was sealed, and the air in the autoclave was replaced three times with nitrogen, with 2 Mpa nitrogen as the initial pressure. Then, the reactor was heated to the desired temperature (220 °C, 240 °C, 260 °C, 280 °C, and 300 °C) for 1 h. Once the reaction was completed, the reactor was cooled to room temperature rapidly. The residue and filtrate were separated by a sand core funnel. The filtrate was evaporated by a rotary evaporator to obtain the ESP, and the residue (ER) was dried using a vacuum drying oven. All experiments were conducted in parallel for three times. The ESP yield (Y_ESP_) was calculated as the ratio of the mass of ESP (m_ESP_) to the mass of dried ash-free base coal (m_NMHC, daf_), i.e., Y_ESP_ = m_ESP_/m_NMHC, daf_.

### 3.4. Catalytic Ethanolysis of BOB

About 20 mL of ethanol, 50 mg of catalyst, and 1 mmol of BOB were added to a 50 mL autoclave, after which the air was replaced with N_2_ for three times and charged with 1 MPa of N_2_. Then, the temperature was increased to 220 °C, 240 °C, 260 °C, and 280 °C and kept for 2 h. After the reaction, the products were separated. Then, the filtrate was analyzed by GC-MS.

### 3.5. Characterization

The physical phase analysis of the synthesized samples was carried out with X-ray diffraction (XRD, Bruker D8 power diffractometer). The scanning was performed between 2θ of 10° and 80° at a scanning rate of 8°/min. The morphology and microstructure of the samples were characterized with scanning electron microscopy (FESEM, Hitachi S-4800). Transmission electron microscopy (TEM) and high-resolution transmission electron microscopy (HRTEM) were performed by using a FEI-TALOS-F200X microscope. Specific surface areas were calculated with Brunauer–Emmtt–Teller (BET) method. Fourier transform infrared spectrometry (FTIR) of the ESP was carried out with a VERTEX-70 infrared (IR) spectrometer using the KBr pellet technique, recorded from 4000 to 400 cm^−1^, and then analyzed using 16 scans. The composition of the ESP was analyzed using a Pegasus 4D^®^/7890B GC-MS. The GC-MS was equipped with a Petro column (50 m × 0.2 mm × 0.5 μm), with an initial column temperature of 60 °C held for 1 min.

## 4. Conclusions

In this work, Fe_2_(MoO_4_)_3_ catalysts with varying dimensions and morphologies were successfully synthesized by different methods, and a series of characterizations were carried out. Four catalysts were applied to the ethanolysis of NMHC, showing good activity; among them, Fe_2_(MoO_4_)_3_ nanorods exhibited the highest catalytic activity. The yield of Fe_2_(MoO_4_)_3_ nanorod-catalyzed ethanolysis reached 47.68% at 260 °C, due to the fact that this catalyst maximized the exposure of the active (100) facets. The GC-MS results of the ESP from the catalytic ethanolysis showed that the amounts of esters, ethers, phenols, and ketones increased, while those of aliphatic hydrocarbons, aromatic hydrocarbons, and heteroatomic compounds decreased compared the corresponding quantities without a catalyst. This indicated that the Fe_2_(MoO_4_)_3_ nanorods catalysts not only significantly increased the ethanolysis yield, but also improved the product distribution. Moreover, the FTIR data were processed by split-peak fitting, and the results indicated that the carbon-oxygen bonds in the coal were broken during catalytic ethanolysis. It was also further verified using BOB as a model compound that the Fe_2_(MoO_4_)_3_ nanorods effectively broke the C-O bond of BOB at 260 °C. The results showed that the high-activity catalyst significantly increased the ESP yield and improved the content of oxygen-containing compounds. Possible reaction pathways for the ethanolysis of Naomaohu coal were deduced from the analysis of the products and model compounds. In summary, catalytic ethanolysis may be an alternative way for the efficient utilization of lignite, and a high-activity catalyst with a good microstructure will help to drive this process.

## Figures and Tables

**Table 1:** Esters detected in the ESP_NC_ and ESP_FMO-2_.

Retention Time/s	Name	Molecular Formula	Relative Content/(wt%)	
			ESP_NC_	ESP_FMO-2_
983.3	methyl 2-hydroxy-2-methylbutanoate	C_6_H_12_O_3_		1.51
1268.7	ethyl 3-methylpentanoate	C_8_H_16_O_2_		0.27
1407.5	ethyl hex-2-enoate	C_8_H_14_O_2_		1.04
1429	ethyl hexanoate	C_8_H_16_O_2_	0.16	1.17
1430.5	isobutyl methacrylate	C_8_H_14_O_2_		0.24
1442.9	ethyl (E)-hex-3-enoate	C_8_H_14_O_2_	0.48	0.44
1444	ethyl hex-4-enoate	C_8_H_14_O_2_		0.51
1448.8	prenyl isobutyrate	C_9_H_16_O_2_	0.18	
1542.8	γ-Caprolactone	C_6_H_10_O_2_	0.45	
1600	ethyl hex-2-enoate	C_8_H_14_O_2_	1.01	6.52
1730.4	4-methyloxan-2-one	C_6_H_10_O_2_		2.16
1804.9	ethyl hept-6-enoate	C_9_H_16_O_2_		0.20
1859.5	ethyl heptanoate	C_9_H_18_O_2_	0.69	0.93
1929.2	ethyl (R)-2-(hydroxymethyl)butanoate	C_7_H_14_O_3_		0.33
2026.3	ethyl octanoate	C_10_H_20_O_2_		0.25
2147.8	diethyl succinate	C_8_H_14_O_4_	1.15	0.29
2153.7	ethyl benzoate	C_9_H_10_O_2_		0.47
2238.5	ethyl oct-7-enoate	C_10_H_18_O_2_	0.19	0.39
2272.2	ethyl (Z)-oct-3-enoate	C_10_H_18_O_2_		0.81
2291.3	ethyl octanoate	C_10_H_20_O_2_	1.98	1.75
2479.2	ethyl 2-octenoate	C_10_H_18_O_2_		0.06
2575.3	diethyl glutarate	C_9_H_16_O_4_	0.15	
2615	ethyl 3-methylbenzoate	C_10_H_12_O_2_	0.25	0.28
2661.8	ethyl non-8-enoate	C_11_H_20_O_2_	0.39	0.20
2709	ethyl nonanoate	C_11_H_22_O_2_	3.16	1.76
3109	ethyl decanoate	C_12_H_24_O_2_	2.05	1.88
3402.7	ethyl 3-hydroxybenzoate	C_9_H_10_O_3_	0.19	
3486.9	ethyl undecanoate	C_13_H_26_O_2_	2.22	
3672.5	ethyl dodecanoate	C_14_H_28_O_2_	1.89	1.89
3714.5	4,4,7a-trimethyl-5,6,7,7a-tetrahydrobenzofuran-2(4H)-one	C_11_H_16_O_2_	0.20	
4430.7	diethyl decanedioate	C_14_H_26_O_4_	0.08	
5020.9	ethyl (E)-hept-3-enoate	C_9_H_16_O_2_	0.24	
5657.1	ethyl undecanoate	C_13_H_26_O_2_	0.22	0.15
6621.4	ethyl 8-methylnonanoate	C_12_H_24_O_2_		3.45
7050.2	ethyl icosanoate	C_22_H_44_O_2_	5.43	7.84
7449.3	ethyl docosanoate	C_24_H_48_O_2_	3.01	

**Table 2:** Phenols detected in the ESP_NC_ and ESP_FMO-2_.

Retention Time/s	Name	Molecular Formula	Relative Content/(wt%)	
			ESP_NC_	ESP_FMO-2_
1320.5	phenol	C_6_H_6_O	2.89	2.56
1636	o-cresol	C_7_H_8_O	0.77	0.78
1720	p-cresol	C_7_H_8_O	1.65	1.55
1870.5	2,6-dimethylphenol	C_8_H_10_O	0.37	0.12
1998.1	2-ethylphenol	C_8_H_10_O	2.54	2.12
2046.7	2,4-dimethylphenol	C_8_H_10_O		0.50
2054.3	2,5-dimethylphenol	C_8_H_10_O	0.37	
2055.2	2,3-dimethylphenol	C_8_H_10_O		0.40
2063.8	2-ethoxyphenol	C_8_H_10_O_2_		0.19
2119.2	4-ethylphenol	C_8_H_10_O		1.30
2128.8	3-ethylphenol	C_8_H_10_O	0.30	
2172.2	2,6-dimethylphenol	C_8_H_10_O		0.06
2206.3	2-ethyl-6-methylphenol	C_9_H_12_O	3.17	4.60
2233.9	2,4-dimethylphenol	C_8_H_10_O	0.07	
2298.9	2,4,6-trimethylphenol	C_9_H_12_O	0.47	0.29
2362.6	2-propylphenol	C_9_H_12_O	0.13	0.15
2453.1	3-ethyl-5-methylphenol	C_9_H_12_O		0.64
2512.5	5-isopropyl-2-methylphenol	C_10_H_14_O	0.71	1.02
2516.5	4-isopropylphenol	C_9_H_12_O	0.22	
2608.1	2-ethyl-4,5-dimethylphenol	C_10_H_14_O	0.74	0.33
2740.2	2,5-diethylphenol	C_10_H_14_O	0.36	0.84
2786.7	2,6-dimethylbenzene-1,4-diol	C_8_H_10_O_2_		0.09
2925.5	4-butylphenol	C_10_H_14_O		0.15
2947.2	2-ethyl-5-propylphenol	C_11_H_16_O		0.20
3186.8	2-(tert-butyl)-4-ethylphenol	C_12_H_18_O		0.29
3458.6	2,6-diisopropylphenol	C_12_H_18_O	0.20	0.11
3476.7	2-isopropyl-5-methylbenzene-1,4-diol	C_13_H_26_O_2_	0.22	
3477.3	4-methoxy-2,3,6-trimethylphenol	C_10_H_14_O_2_		0.31
3612.2	3-(tert-butyl)-4-methoxyphenol	C_11_H_16_O_2_	0.08	0.42
3714.7	2-(tert-butyl)-4-methoxyphenol	C_11_H_16_O_2_		0.07

**Table 3:** Catalytic ethanolysis of BOB at different temperatures.

Temperature (°C)	Conversion (%)	Selectivity (%)				
	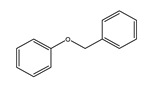	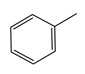	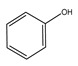	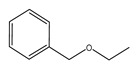	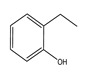	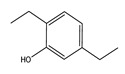
220	46.77	45.80	34.14	16.21	3.84	0
240	92.39	45.27	22.35	10.52	18.35	3.51
260	100	43.59	11.64	6.30	25.00	13.47
280	100	41.68	8.95	6.19	24.47	18.71

**Table 4:** Proximate and ultimate analysis of NMHC.

Proximate Analysis (wt%)			Ultimate Analysis (wt%, daf)				H/C
M_ad_	A_d_	V_daf_	C	H	N	O_diff_	
7.34	4.73	49.86	63.38	4.63	0.82	26.79	0.8766

_ad_: air-dry basis; _daf_: dry and ash-free basis; _diff_: by difference.

## Data Availability

The data are contained within the article.

## Supplementary Materials

The following supporting information can be downloaded at: https://www.mdpi.com/article/10.3390/molecules28186595/s1, Table S1: The specific surface area and average pore size and pore volume of the Fe_2_(MoO_4_)_3_ samples. Table S2: Ketones detected in the ESP_NC_ and ESP_FMO-2_. Table S3: Alcohols detected in the ESP_NC_ and ESP_FMO-2_. Table S4: Aromatic hydrocarbons detected in the ESP_NC_ and ESP_FMO-2_. Table S5: Aliphatic hydrocarbons detected in the ESP_NC_ and ESP_FMO-2_. Table S6: Acids detected in the ESP_NC_ and ESP_FMO-2_. Table S7: Ethers detected in the ESP_NC_ and ESP_FMO-2_. Table S8: Aldehydes detected in the ESP_NC_ and ESP_FMO-2_. Table S9: Heteroatom compounds detected in the ESP_NC_ and ESP_FMO-2_. Table S10: Oxygenated functional group region fitting peak information for NMHC. Table S11: Oxygenated functional group region fitting peak information for ERNC. Table S12: Oxygenated functional group region fitting peak information for ER_FMO-2_. Figure S1. NH_3_-TPD profile of FMO-2. Figure S2: FTIR spectrum of NMHC and ER. Figure S3: FTIR spectrum and related curves of oxygen-containing functional groups in NMHC. Figure S4: FTIR spectrum and related curves of oxygen-containing functional groups in ER_NC_. Figure S5: FTIR spectrum and related curves of oxygen-containing functional groups in ER_FMO-2_.

## Sample Availability

Samples of the compounds are available from the authors.
