# Supplementary material for: Insights into the Relationship between the Microstructure and the Catalytic Behavior of Fe2(MoO4)3 during the Ethanolysis of Naomaohu Coal
Source: Molecules. 2023 Sep 13;28(18):6595. doi: 10.3390/molecules28186595 (PMC10535724; doi:10.3390/molecules28186595)

Supplementary Materials

Insights into the Relationship between the Microstructure and the Catalytic Behavior of $Fe_2(MoO_4)_3$ during the Ethanolysis of Naomaohu Coal

Ting Liu *,†, Xuesong Sun †, Yakun Tang, Yue Zhang, Jingmei Liu, Xiaodong Zhou, Xiaohui Li and Lang Liu *

State Key Laboratory of Chemistry and Utilization of Carbon Based Energy Resources, College of Chemistry, Xinjiang University, Urumqi 830017, China; 17864301835@163.com (X.S.); yktang@xju.edu.cn (Y.T.); yuezhang@xju.edu.cn (Y.Z.); liujm@xju.edu.cn (J.L.); xd_zhou@126.com (X.Z.); lxhui0611@163.com (X.L.)

* Correspondence: liut@xju.edu.cn (T.L.); liulang@xju.edu.cn (L.L.)

† These authors contributed equally to this work.

Content

Table S1. The specific surface area, average pore size and pore volume of $Fe_2(MoO_4)_3$ samples.

Table S2. Ketones detected in the ESP_{NC} and ESP_{FMO-2}.

Table S3. Alcohols detected in the ESP_{NC} and ESP_{FMO-2}.

Table S4. Aromatic hydrocarbons detected in the ESP_{NC} and ESP_{FMO-2}.

Table S5. Aliphatic hydrocarbons detected in the ESP_{NC} and ESP_{FMO-2}.

Table S6. Acids detected in the ESP_{NC} and ESP_{FMO-2}.

Table S7. Ethers detected in the ESP_{NC} and ESP_{FMO-2}.

Table S8. Aldehydes detected in the ESP_{NC} and ESP_{FMO-2}.

Table S9. Heteroatom compounds detected in the ESP_{NC} and ESP_{FMO-2}.

Table S10. Oxygenated functional group region fitting peak information of NMHC.

Table S11. Oxygenated functional group region fitting peak information of the ER_{NC}.

Table S12. Oxygenated functional group region fitting peak information of the ER_{FMO-2}.

Figure S1. NH_3-TPD profile of FMO-2.

Figure S2. FTIR spectrum of NMHC and ER.

Figure S3. FTIR spectrum and its curvesof oxygen-containing functional groups in NMHC.

Figure S4. FTIR spectrum and its curvesof oxygen-containing functional groups in ER_{NC}.

Figure S5. FTIR spectrum and its curvesof oxygen-containing functional groups in ER_{FMO-2}.

Table S1

The specific surface area, average pore size and pore volume of $Fe_2(MoO_4)_3$ samples.

$Fe_2(MoO_4)_3$	S_{BET} (m^2/g)	Average pore size (nm)	Pore volume (cm^3/g)
FMO-1	21.0127	17.2056	0.0903
FMO-2	14.0543	7.4407	0.0261
FMO-3	12.9181	15.7431	0.0508
FMO-4	17.6475	17.6666	0.0779

Table S2

Ketones detected in the ESP_{NC} and ESP_{FMO-2}.

Retention time/s	Name	Molecular Formula	Relative content/ (wt%)	
			ESP_{NC}	ESP_{FMO-2}
1380.1	bicyclo[3.1.1]heptan-2-one	$C_7H_{10}O$		0.14
1543.9	5-butyldihydrofuran-2(3H)-one	$C_8H_{14}O_2$		0.48
1546.1	2,3-dimethylcyclopent-2-en-1-one	$C_7H_{10}O$	0.46	
1747.2	nonan-4-one	$C_9H_{18}O$		0.28
1777.6	1-(2-methylcyclopent-1-en-1-yl)ethan-1-one	$C_8H_{12}O$		0.08
1833.9	5-methyl-3-methylenehex-5-en-2-one	$C_8H_{12}O$		0.17
1924.8	4-propylcyclopent-4-ene-1,3-dione	$C_8H_{10}O_2$		0.16
2069.6	2,3-dimethylcyclopent-2-en-1-one	$C_7H_{10}O$		0.19
2152.8	2,2-dihydroxy-1-phenylethan-1-one	$C_8H_8O_3$	0.24	
2439.4	1-(2-hydroxyphenyl)ethan-1-one	$C_8H_8O_2$	0.15	
2561.6	astaxanthin	$C_{40}H_{52}O_4$		0.19
2775.2	(3,5-Dimethoxyphenyl)(4-methoxyphenyl)methanone	$C_{16}H_{16}O_4$		0.16

Table S3

Alcohols detected in the ESP_{NC} and ESP_{FMO-2}.

Retention time/s	Name	Molecular Formula	Relative content/ (wt%)	
			ESP_{NC}	ESP_{FMO-2}
747.2	3-methylpent-4-en-1-ol	$C_6H_{12}O$		0.61
845	3-methylpentan-1-ol	$C_6H_{14}O$		1.58
864	3-Hexen-1-ol	$C_6H_{12}O$		0.49
879.4	cis-3-Hexen-1-ol	$C_6H_{12}O$		0.72
932.6	3-(oxiran-2-yl)propan-1-ol	$C_5H_{10}O_2$		0.24
1840.8	2-methyl-1-phenylpropan-1-ol	$C_{10}H_{14}O$		0.10
2011	o-tolylmethanol	$C_8H_{10}O$		0.11
2575.9	Zeaxanthin	$C_{40}H_{56}O_2$		0.11
2884.2	(4-(tert-butyl)phenyl)methanol	$C_{11}H_{16}O$	0.63	0.47
5063	(9Z,12Z)-octadeca-9,12-dien-1-ol	$C_{18}H_{34}O$	0.87	

Table S4

Aromatic hydrocarbons detected in ESP_{NC} and ESP_{FMO-2}.

Retention time/s	Name	Molecular Formula	Relative content/ (wt%)	
			ESP_{NC}	ESP_{FMO-2}
2071.1	2-methyl-2,3-dihydro-1H-indene	$C_{10}H_{12}$		0.10
2114.9	but-2-en-1-ylbenzene	$C_{10}H_{12}$		0.15
2160.4	1,2,3,4-tetrahydronaphthalene	$C_{10}H_{12}$	0.88	0.90
2242.9	azulene	$C_{10}H_8$	10.98	0.82
2282.8	2,2-dimethyl-2,3-dihydro-1H-indene	$C_{11}H_{14}$		0.11
2324.8	1,1-dimethyl-2,3-dihydro-1H-indene	$C_{11}H_{14}$		0.11
2679.8	pent-2-en-2-ylbenzene	$C_{11}H_{14}$		0.10
2714.2	1-ethylidene-1H-indene	$C_{11}H_{10}$		0.28
2778.6	1-methylnaphthalene	$C_{11}H_{10}$	4.01	0.17
3035.6	1,1'-biphenyl	$C_{12}H_{10}$	0.22	
3112.2	2-ethylnaphthalene	$C_{12}H_{12}$	0.32	
3157.1	1,8-dimethylnaphthalene	$C_{12}H_{12}$	0.27	
3164.2	1,4-dimethylnaphthalene	$C_{12}H_{12}$	0.53	0.11
3227.7	1,7-dimethylnaphthalene	$C_{12}H_{12}$	0.69	0.11
3602	1,1,2,3,3-pentamethylindan	$C_{14}H_{20}$	0.19	
3802.9	9H-fluorene	$C_{13}H_{10}$	1.04	
4145.4	benzo[m]tetraphene	$C_{22}H_{14}$	0.59	
4225	9-methyl-9H-fluorene	$C_{14}H_{12}$	0.08	
4455	9-methylene-9H-fluorene	$C_{14}H_{10}$		0.10
4456	phenanthrene	$C_{14}H_{10}$	6.95	
4818	2-methylphenanthrene	$C_{15}H_{12}$	0.68	
5044.2	nonylbenzene	$C_{15}H_{24}$	0.24	
5293	fluoranthene	$C_{16}H_{10}$	0.40	
5350.7	1-ethyl-4-methylbenzene	C_9H_{12}	0.19	
5425.3	naphtho[7,8,1,2,3-nopqr]tetraphene	$C_{22}H_{12}$	2.97	
5436.1	pyrene	$C_{16}H_{10}$	0.66	
6303.2	triphenylene	$C_{18}H_{12}$	0.25	
7516.4	indeno[1,2,3-gh]tetraphene	$C_{24}H_{14}$	0.97	
7517.7	dibenzo[f,pqr]tetraphene	$C_{24}H_{14}$	1.53	

Table S5

Aliphatic hydrocarbons detected in the ESP_{NC} and ESP_{FMO-2}.

Retention time/s	Name	Molecular Formula	Relative content/ (wt%)	
			ESP_{NC}	ESP_{FMO-2}
834.3	2,3-dimethylhexane	C_8H_{18}		0.24
921.8	ethylcyclobutane	C_6H_{12}		1.66
1544.8	1,2-dipropylcycloprop-1-ene	C_9H_{16}		0.14
1546.8	prop-1-en-2-ylcyclopropane	C_6H_{10}		0.43
1950	5,7-dimethylundecane	$C_{13}H_{28}$		0.17
2068.4	propan-2-ylidenecyclopentane	C_8H_{14}	0.13	
2329.2	1,2,4,4-tetramethylcyclopent-1-ene	C_9H_{16}		0.19
2382.1	2,9-dimethyldecane	$C_{12}H_{26}$	0.28	
2382.9	tridecane	$C_{13}H_{28}$		0.39
2799	2-methylnonane	$C_{10}H_{22}$	0.56	
2799.6	tetradecane	$C_{14}H_{30}$		0.58
3195.4	undecane	$C_{11}H_{24}$	0.72	
3571	heptadecane	$C_{17}H_{36}$		1.46
3925.7	hexadecane	$C_{16}H_{34}$	1.66	0.56
4582.1	2-methyldodecane	$C_{13}H_{28}$		0.67
4885.6	2-methylundecane	$C_{12}H_{26}$	0.99	1.11
5062.9	hexadec-1-yne	$C_{16}H_{30}$		0.46
5451.5	2,6,10-trimethyldodecane	$C_{15}H_{32}$	0.35	
5715.8	nonadecane	$C_{19}H_{40}$	1.74	1.10
5968.6	heptacosane	$C_{27}H_{56}$	1.08	
6883.9	2-methylnonadecane	$C_{20}H_{42}$	0.77	0.39
7292.4	3,7-diethyl-5-propylnonane	$C_{26}H_{54}$	0.63	

Table S6

Acids detected in the ESP_{NC} and ESP_{FMO-2}.

Retention time/s	Name	Molecular Formula	Relative content/ (wt%)	
			ESP_{NC}	ESP_{FMO-2}
1537.2	2-hydroxy-2-phenylacetic acid	$C_8H_8O_3$		0.21
2161.1	maleic acid	$C_4H_4O_4$		0.24
2451.7	3-(2,4-dimethoxyphenyl)acrylic acid	$C_{11}H_{12}O_4$		0.08

Table S7

Ethers detected in the ESP_{NC} and ESP_{FMO-2}.

Retention time/s	Name	Molecular Formula	Relative content/ (wt%)	
			ESP_{NC}	ESP_{FMO-2}
554.8	1,1-diethoxyethane	$C_6H_{14}O_2$		1.38
1072.5	1,1-diethoxybutane	$C_8H_{18}O_2$		2.82
1117.5	1-(1-ethoxyethoxy)butane	$C_8H_{18}O_2$		0.36
1587.9	(Z)-1,1-diethoxyhex-3-ene	$C_{10}H_{20}O_2$		0.52
1761	2,4-dimethylfuran	C_6H_8O		0.07
1901.3	1,1-diethoxy-2-methylpropane	$C_8H_{18}O_2$		9.68
2234.6	1-methoxy-4-methylbenzene	$C_8H_{10}O$		0.14
2945.9	carvacrol methyl ether	$C_{11}H_{16}O$	0.35	
3201.3	1-ethoxy-2-isopropylbenzene	$C_{11}H_{16}O$		0.08
3819.2	1,4-dimethoxy-2,3,5,6-tetramethylbenzene	$C_{12}H_{18}O_2$		0.61

Table S8

Aldehydes detected in the ESP_{NC} and ESP_{FMO-2}.

Retention time/s	Name	Molecular Formula	Relative content/ (wt%)	
			ESP_{NC}	ESP_{FMO-2}
1232.8	benzaldehyde	C_7H_6O		0.19
1611.6	octa-2,4,6-trienal	$C_8H_{10}O$		0.20
1689.9	3-methylbenzaldehyde	C_8H_8O	0.26	0.72
1749	4-methylbenzaldehyde	C_8H_8O		0.36
2632	(2S,3R,4R,5S)-2,3,4,5-tetrahydroxyhexanal	$C_6H_{12}O_5$		0.21
3089.9	6-methyl-4-oxo-4H-chromene-3-carbaldehyde	$C_{11}H_8O_3$		0.17

Table S9

Heteroatom compounds detected in the ESP_{NC} and ESP_{FMO-2}.

Retention time/s	Name	Molecular Formula	Relative content/ (wt%)	
			ESP_{NC}	ESP_{FMO-2}
634.9	thiourea	CH_4N_2S		0.35
682.3	triethylamine	$C_6H_{15}N$	0.95	
747.1	2-methylpyridine	C_6H_7N	5.15	0.93
828	L-isoleucyl-L-cysteine	$C_9H_{18}N_2O_3S$		0.27
886	3-Picoline	C_6H_7N	0.94	0.72
982.7	L-valyl-L-serine	$C_8H_{16}N_2O_4$	0.32	
1031	9-Hydroxy Topiramate	$C_{12}H_{21}NO_9S$		0.10
1100.9	4-amino-6-(ethylamino)-1,3,5-triazin-2(5H)-one	$C_5H_9N_5O$		0.25
1408.2	2-propylpyridine	$C_8H_{11}N$	0.36	0.53
1471.8	ethyl(propyl)sulfane	$C_5H_{12}S$		0.17
1499.6	3-methylbutan-2-yl 2,2,2-trifluoroacetate	$C_7H_{11}F_3O_2$		0.14
1523.6	5-ethyl-2-methylpyridine	$C_8H_{11}N$	0.50	0.43
1563.3	2,3,5-trimethylpyridine	$C_8H_{11}N$	0.11	0.26
1662.5	4-propylpyridine	$C_8H_{11}N$	0.42	0.81
1729.8	2-amino-2-cyanoacetic acid	$C_3H_4N_2O_2$	0.46	
1919.2	cycloheptanamine	$C_7H_{15}N$		0.28
2208.4	L-valyl-L-isoleucine	$C_{11}H_{22}N_2O_3$		0.20
2301.3	4-(pentan-3-yl)pyridine	$C_{10}H_{15}N$		0.26
2305.3	2,3-dihydro-1H-inden-2-amine	$C_9H_{11}N$		0.13
2384.2	methyl 4-nitrohexanoate	$C_7H_{13}NO_4$		0.12
2660	4-methylbenzohydrazide	$C_8H_{10}N_2O$		0.19
2695.7	4-(ethylthio)butan-2-one	$C_6H_{12}OS$		0.18
3434.4	zectran	$C_{12}H_{18}N_2O_2$		0.09
3537.1	Levomefolic acid	$C_{20}H_{25}N_7O_6$	0.08	
4365.9	dibenzo[b,d]thiophene	$C_{12}H_8S$	0.18	
5715.7	butyl decyl sulfite	$C_{14}H_{30}O_3S$	1.04	
6211	butyl dodecyl sulfite	$C_{16}H_{34}O_3S$	0.97	
6394.6	L-valylglycine	$C_7H_{14}N_2O_3$		0.10
6444.3	pentyl undecyl sulfite	$C_{16}H_{34}O_3S$		0.55
6964.4	oleamide	$C_{18}H_{35}NO$	0.67	0.14
7092	hexyl tridecyl sulfite	$C_{19}H_{40}O_3S$	0.35	

Table S10

Oxygenated functional group region fitting peak information of NMHC.

No	Position	Assignment	Percentage
1	1069	Alkyl ethers	1.8%
2	1119	C-O sec alcohols	3.3%
3	1169	C-O phenols ethers	6.1%
4	1215	C-O phenols ethers	5.6%
5	1257	C-O in aryl ethers	6.1%
6	1298	C-O in aryl ethers	6.2%
7	1338	C-O in aryl ethers	5.1%
8	1378	CH_3-Ar, R	6.3%
9	1419	CH_3-Ar, R	6.4%
10	1456	$-CH_3$ $-CH_2$	7.9%
11	1500	Aromatic C=C	5.7%
12	1545	Aromatic C=C	6.4%
13	1581	Aromatic C=C	8.3%
14	1617	Aromatic C=C	10.1%
15	1654	Conjugated C=O	7.4%
16	1701	Carboxyl acids	5.4%
17	1736	Aryl esters	2.0%
18	1752	Alicyclic C=O	0

Table S11

Oxygenated functional group region fitting peak information of the ER_{NC}.

No.	Position	Assignment	Percentage
1	1047	Alkyl ethers	2.2%
2	1109	C-O sec alcohols	4.6%
3	1166	C-O phenols ethers	6.8%
4	1213	C-O phenols ethers	6.2%
5	1255	C-O in aryl ethers	6.2%
6	1293	C-O in aryl ethers	5.8%
7	1330	C-O in aryl ethers	4.8%
8	1373	CH_3-Ar, R	6.5%
9	1417	CH_3-Ar, R	5.7%
10	1453	$-CH_3$ $-CH_2$	8.5%
11	1494	Aromatic C=C	4.0%
12	1536	Aromatic C=C	4.3%
13	1575	Aromatic C=C	8.1%
14	1613	Aromatic C=C	9.9%
15	1650	Conjugated C=O	6.2%
16	1700	Carboxyl acids	5.3%
17	1727	Aryl esters	4.6%
18	1786	Alicyclic C=O	0.4%

Table S12

Oxygenated functional group region fitting peak information of ER_{FMO-2}.

No.	Position	Assignment	Percentage
1	1026	Alkyl ethers	1.7%
2	1091	C-O sec alcohols	3.2%
3	1134	C-O phenols ethers	3.4%
4	1176	C-O phenols ethers	5.1%
5	1230	C-O Phenols ethers	5.7%
6	1253	C-O in aryl ethers	6.0%
7	1296	C-O in aryl ethers	5.9%
8	1323	C-O in aryl ethers	5.4%
9	1373	CH_3-Ar, R	6.7%
10	1415	CH_3-Ar, R	7.1%
11	1454	-CH_3 -CH_2	9.1%
12	1492	Aromatic C=C	3.7%
13	1535	Aromatic C=C	5.1%
14	1573	Aromatic C=C	8.8%
15	1604	Aromatic C=C	9.8%
16	1651	Conjugated C=O	4.9%
17	1701	Carboxyl acids	4.6%
18	1728	Aryl esters	0

Figure S1. NH_3-TPD profile of FMO-2.

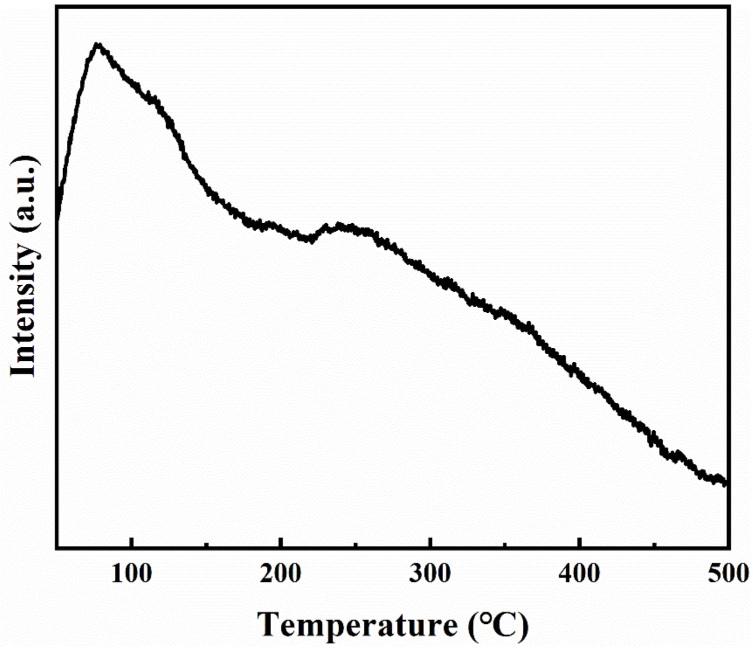

Figure S2. FTIR spectrum of NMHC and ER.

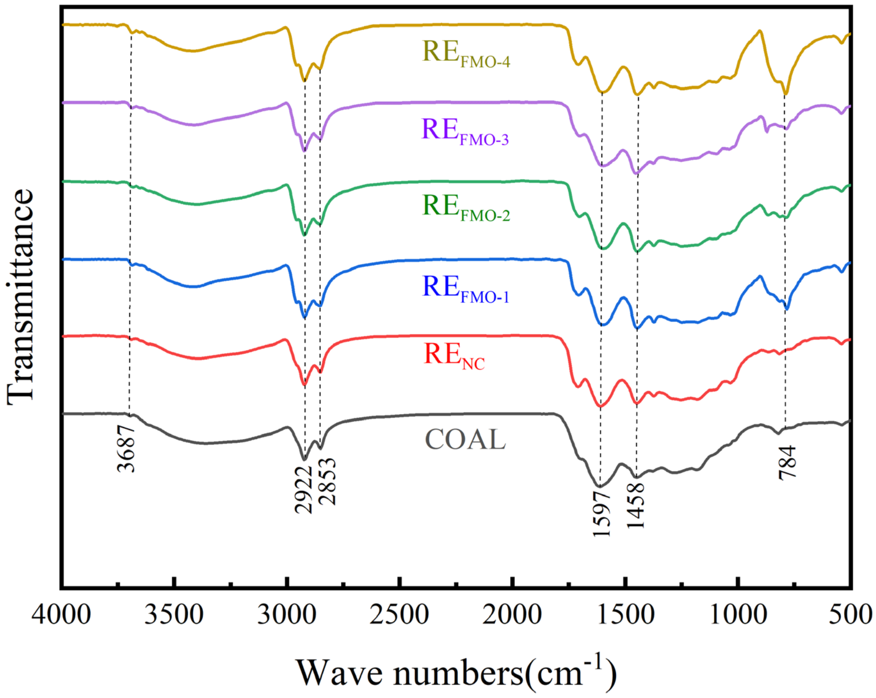

Figure S3. FTIR spectrum and its curvesof oxygen-containing functional groups in NMHC.

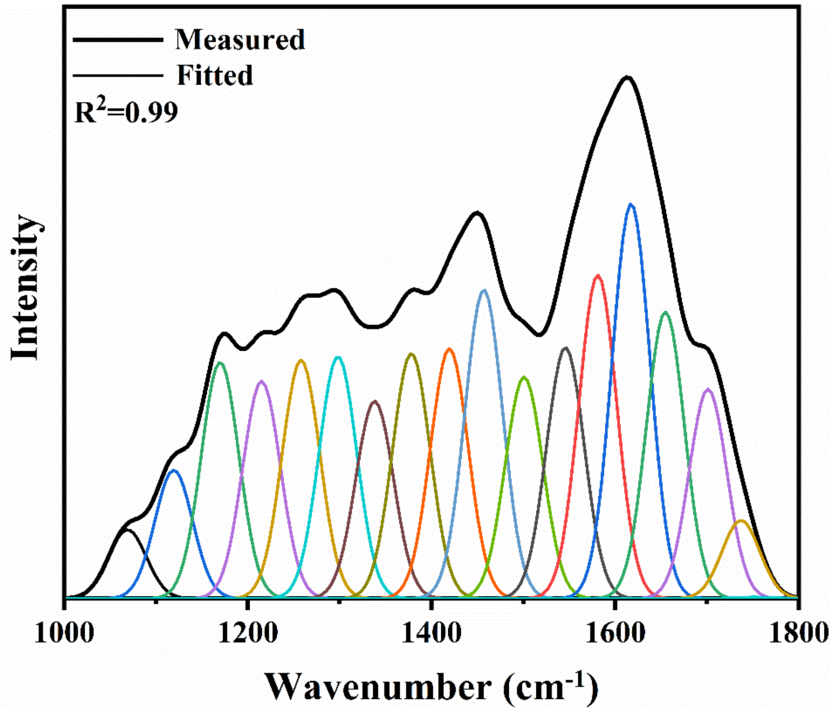

Figure S4. FTIR spectrum and its curvesof oxygen-containing functional groups in ER_{NC}.

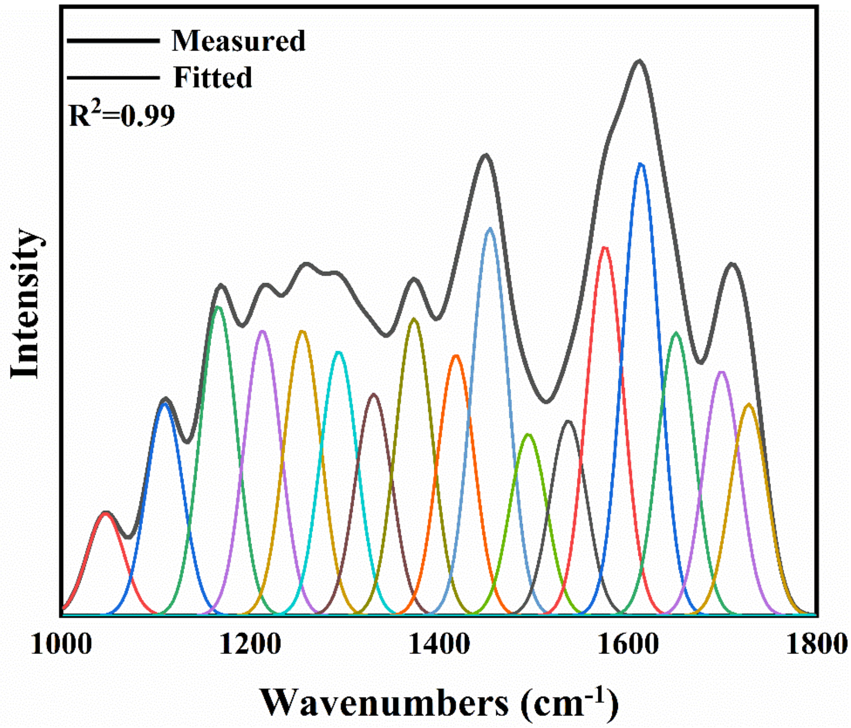

Figure S5 FTIR spectrum and its curvesof oxygen-containing functional groups in ER_{FMO-2}.

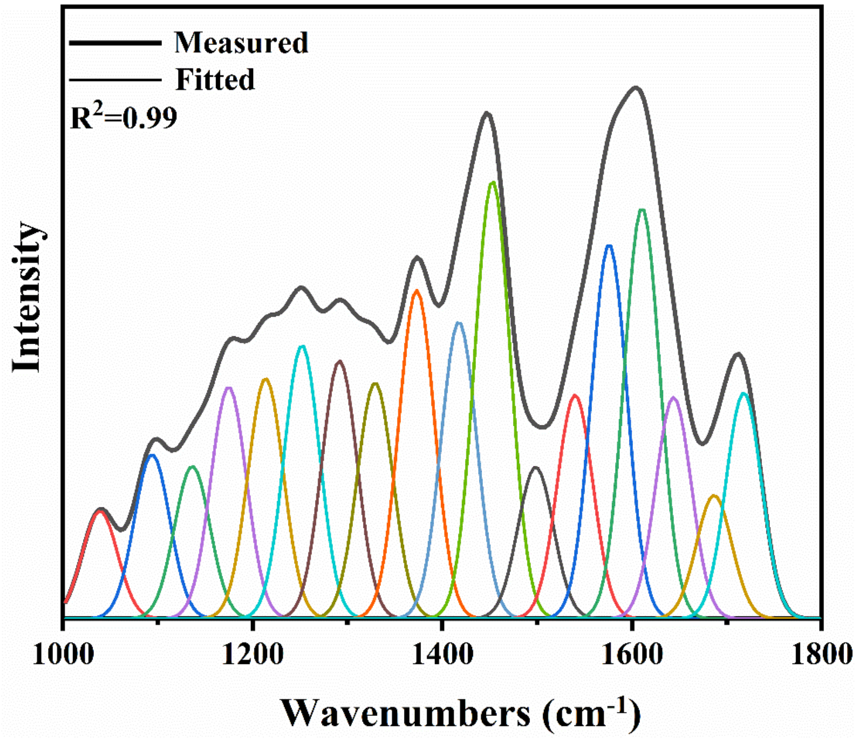

Supplement: Supplementary file 1 [file molecules-28-06595-s001.zip › molecules-2482324-supplementary.pdf]
